# Three-Dimensional Bioprinting for Cartilage Tissue Engineering: Insights into Naturally-Derived Bioinks from Land and Marine Sources

**DOI:** 10.3390/jfb13030118

**Published:** 2022-08-12

**Authors:** Marta Anna Szychlinska, Fabio Bucchieri, Alberto Fucarino, Alfredo Ronca, Ugo D’Amora

**Affiliations:** 1 Department of Biomedicine, Neuroscience and Advanced Diagnostics, University of Palermo, 90127 Palermo, Italy; 2 Institute of Polymers, Composites and Biomaterials, National Research Council, 80125 Naples, Italy

**Keywords:** 3D bioprinting, additive manufacturing, bioink, cartilage tissue engineering, land sources, marine sources

## Abstract

In regenerative medicine and tissue engineering, the possibility to: (I) customize the shape and size of scaffolds, (II) develop highly mimicked tissues with a precise digital control, (III) manufacture complex structures and (IV) reduce the wastes related to the production process, are the main advantages of additive manufacturing technologies such as three-dimensional (3D) bioprinting. Specifically, this technique, which uses suitable hydrogel-based bioinks, enriched with cells and/or growth factors, has received significant consideration, especially in cartilage tissue engineering (CTE). In this field of interest, it may allow mimicking the complex native zonal hyaline cartilage organization by further enhancing its biological cues. However, there are still some limitations that need to be overcome before 3D bioprinting may be globally used for scaffolds’ development and their clinical translation. One of them is represented by the poor availability of appropriate, biocompatible and eco-friendly biomaterials, which should present a series of specific requirements to be used and transformed into a proper bioink for CTE. In this scenario, considering that, nowadays, the environmental decline is of the highest concerns worldwide, exploring naturally-derived hydrogels has attracted outstanding attention throughout the scientific community. For this reason, a comprehensive review of the naturally-derived hydrogels, commonly employed as bioinks in CTE, was carried out. In particular, the current state of art regarding eco-friendly and natural bioinks’ development for CTE was explored. Overall, this paper gives an overview of 3D bioprinting for CTE to guide future research towards the development of more reliable, customized, eco-friendly and innovative strategies for CTE.

## 1. Introduction

Over recent years, additive manufacturing technologies, such as three-dimensional (3D) bioprinting, have emerged as a powerful tool for the custom-made 3D objects’ fabrication, through a controlled deposition of different types of materials, overwhelming the classical limitations of the traditional strategies. It represents a revolutionary technology widely applied to several areas, including the food industry, textile, fashion and medicine [1,2]. In the biomedical area, in particular, 3D bioprinting has received significant attention in the field of regenerative medicine and tissue engineering, since it allows the digitally assisted manufacturing of highly organized native mimicking tissues [3]. Specifically, in cartilage tissue engineering (CTE), 3D bioprinting has allowed the recapitulating of the complex native zonal structure of hyaline cartilage by extrusion/injection-based processes and the controlled deposition of cell-seeded hydrogel-based bioinks [4,5,6]. Articular cartilage has a complex load-bearing tissue organization, whose main function is covering the bone surface in diarthrodial joints. Differently from the majority of tissues, cartilage is basically an avascular, aneural and hypocellular tissue. In particular, the absence of vascularization can significantly prevent the capability of injured cartilage to heal itself once damaged [7]. Limitations of available therapeutic treatments, such as arthroplasty and microfractures, have prompted the field of CTE, based on the synergistic use of stem cells and engineered 3D biodegradable scaffolds, suitably functionalized with bioactive molecules and able to promote cartilage tissue repair and regeneration [8,9,10,11,12]. Bearing this in mind, the additive, layer-by-layer nature of 3D bioprinting may allow the generation of complex cell-seeded structures through rapid manufacturing with high-precision and control over different parameters (i.e., porosity, size and shape, distribution of cells and use of biochemical factors and bioactive materials), fabricating the next generation of customized scaffolds [13,14]. However, despite the deep knowledge achieved in biomaterials science and engineering, there are still some limitations to be overcome before 3D bioprinting may be globally employed for CTE products’ development and their clinical translational use. One of the main limitations is the poor availability of appropriate, biocompatible and eco-friendly biomaterials [15]. In particular, the ideal biomaterial for this purpose should present a series of specific characteristics to be used and transformed into an appropriate bioink. Indeed, the 3D bioprinting is mainly based on the extrusion/injection of a naturally-derived polymeric bioink, in the form of a hydrogel, in the liquid state, which has to maintain its shape over time, allowing the development of viable customized biological structures in a post-processing phase. For this reason, bioinks should (a) be extrudable/injectable through a thin needle, (b) be characterized by a shear thinning behavior, (c) have a suitable elasticity to maintain a 3D highly interconnected porous structure with a good shaped fidelity, (d) present optimized diffusivity of oxygen (O_2_)/nutrients, (e) present controlled biodegradability and (f) support cell growth. Moreover, considering that bioinks embed living cells, they need to be printed at lower working temperatures, requesting mild, cell-friendly crosslinking strategies to maintain the cells being viable and active [16]. In addition to the biological, morphological, mass transport and rheological features, in order to be validated in vivo, bioinks must provide suitable mechanical strength (i.e., suitable Young’s modulus and specific viscoelastic moduli) to recapitulate the macro- and micro-architecture of the native tissue and support de novo tissue formation. Articular cartilage, as supportive structural tissue, is characterized by considerably higher biomechanical features to support the wide range of load bearing and shear forces naturally present in vivo. In particular, it exhibits a dynamic modulus from 0.1 to 3 MPa, in the frequency range of 1–1000 Hz [17]. From this point of view, the pursuit of a bioink, capable of mimicking these mechanical properties, with a specific viscoelastic behavior and the ability to recapitulate the exact cell microenvironment, adds a great level of complexity to the challenge of its perfect selection.

Given the great attention on the environmental decline occurring to date worldwide due to the rapidly growing industry, the request of products obtained starting from renewable and sustainable non-petroleum-based resources is increasing. For this reason, exploring naturally-derived hydrogel biomaterials is attracting outstanding attention from the scientific community. Discovering and promoting the use of these biopolymers, as bioinks and scaffolds’ matrices for CTE, might not only support the sustainable and recycling economy, but also reduce the negative side effects of synthetic materials in biomedical applications. Furthermore, engineered biodegradable naturally-derived polymers may also allow the design and development of 3D printed smart biomolecule delivery devices able to promote in situ tissue repair and regeneration [18].

Therefore, the present review investigates the actual knowledge regarding the most used eco-friendly and naturally-derived hydrogels, as bioinks, for the development of bioactive customized 3D-bioprinted scaffolds for CTE. The review focuses its attention on 3D bioprinting and highlights the main requirements that should be achieved to produce a highly eco-sustainable 3D scaffold for CTE, intending to guide future research, which will capitalize on the potential of biomaterials science, looking toward more reliable bioinks and innovative approaches for this field of interest.

## 2. Naturally-Derived Bioinks for 3D Bioprinting of Cartilage Tissue

**Figure 1:**
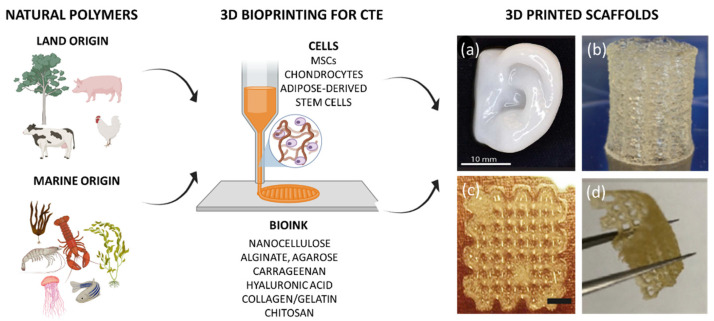
Schematic representation of the main natural sources to obtain bioinks suitable for the design of 3D bioprinted scaffolds for cartilage tissue engineering (CTE). (**a**) A 3D printed human ear with nanofibrillated cellulose/alginate (80:20 *w*/*w*). Adapted with permission from Markstedt et al. [25]. Copyright 2015, American Chemical Society. (**b**) Alginate-di-aldehyde/gelatin 3D printed scaffold. Adapted with permission from Kreller et al. [26]. Copyright 2021, Elsevier (**c**) A 3D printed methacrylated hyaluronic acid scaffold. Adapted with permission from Poldervaart et al. [27]. Copyright 2017, Public Library of Science (**d**) A 3D printed scaffold made of 10% (*w*/*v*) chitosan. Adapted with permission from Sadeghianmaryan et al. [28]. Copyright 2020, Elsevier.

For orthopedic surgeons and patients, the regeneration of cartilage defects has been a huge challenge. In a recent epidemiological study, osteoarthritis affects about 50 million patients and 43.5% of them have stated that it negatively affects their life [19,20]. Actually, cartilage lesions treatment has not been fully exploited. Fortunately, CTE has progressed into the most promising therapeutic strategy for healing cartilage tissue [21]. As already mentioned, to develop an ideal CTE program, it is important to provide well-designed biomaterials that mimic the natural extracellular matrix (ECM) of cartilage with suitable mechanical features. In this scenario, the search for a biomaterial with the appropriate equilibrium of mechanical and biological features is still a big challenge [22]. Furthermore, it is worth noting that to refine a biomaterial into a bioink suitable for 3D printing application, additional requests on the criteria for the selection of the best candidate biomaterial should be taken into consideration. Synthetic materials (i.e., polyethylene glycol, polyvinyl alcohol, pluronic acid-based bioinks) display advantages in terms of mechanical properties, which can also be easily modified to increase, for example, the viscosity and printability. However, their major limitations are represented by their poor bioactivity, high immunogenicity and the risk of preventing de novo tissue development [23,24]. Furthermore, they may present toxic by-products as result of their in vivo degradation. On the contrary, natural materials or biopolymers have played a pivotal role in reproducing cell-friendly microenvironments for bioprinted cells, owing to their unique advantageous properties, namely biocompatibility, non-toxicity and biodegradation. Indeed, naturally-derived bioinks have highlighted a better ability to ensure cell functions if compared to synthetic bioinks. Among the disadvantages, it is worth noting the limited mechanical properties and residence time, which can be modulated by following suitable chemical modifications. Furthermore, natural bioinks can be generally obtained from various renewable resources free of ecological burdens, such as vegetables (land plants and marine algae) and animals from land and marine sources as well. Despite plant-derived bioinks, animal-derived ones have shown improved cell/material interactions. In this context, inspired by biological macromolecules within the ECM, hydrogels, for example, proteins such as collagen (COL) as well as polysaccharides such as cellulose, chitosan (CS), alginate (ALG) and hyaluronic acid (HA) have been commonly employed for CTE. In the following paragraphs, an overview of the mostly employed and potential natural polymers derived from vegetable and animal origins, both from land and marine sources, for the development of 3D-bioprinted CTE scaffolds, is reported (Figure 1).

### 2.1. Three-Dimensional Printing of Principal Vegetable-Derived Hydrogels for CTE

#### 2.1.1. Land Plants: Cellulose and Nanocellulose

Cellulose is the most abundant polymer on Earth. It is a polysaccharide composed of D-glucopyranose linked by β-1,4 glycosidic bonds. It is characterized by three hydroxyl groups (−OH) at C-2, C-3 and C-6 positions, which are responsible for its physical properties. In nature, cellulose is a versatile, structural polymer that provides robust mechanical properties to plant cells, thanks to the hierarchical organization of its natural fibers [29]. A particular cellulose-based extract is nanocellulose (NC). Generally, NC can be classified into three types: cellulose nanocrystals (CNC), nanofibrillated cellulose (NFC) and bacterial cellulose. The attention of the present review will be focused on the first two classes of NC. CNC and NFC are mainly extracted from wood, cotton, algae, potato tuber, and hemp [29]. From a morphological point of view, NC presents a wide array of required properties for 3D bioprinting and tissue engineering. In particular, NFC-based hydrogels show non-Newtonian shear thinning features, with a storage modulus generally higher than the loss modulus at low shear rates [25]. Due to the chemical structure, NC is highly resistant to degradation in the human body. To overcome this problem, the biomaterial could be functionalized with cellulase enzymes to hydrolyze β-1,4 linkages. Cartilage tissue has been successfully bioprinted using NC blends (Table 1) [25,30]. Indeed, so far, no proof of neat NC hydrogels as suitable bioinks has been found. In particular, in several studies, NC has been blended with other natural polymers in order to obtain increased mechanical properties, long-term stability, shape fidelity and structural integrity in the post-processing phase, as summarized in Table 1.

**Figure 2:**
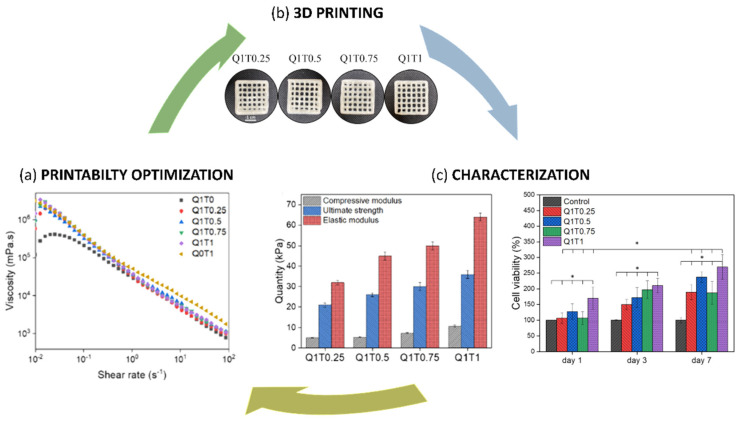
(**a**) Printability Optimization: apparent viscosity versus shear rate, highlighting the shear thinning behavior, (**b**) representative images of 3D printed Quince Seed Mucilage/Nanofibrillated Cellulose (QSM/NFC) scaffolds, (**c**) Mechanical (**left**) and biological (**right**) characterization. Q1T0.25, Q1T0.5, Q1T0.75, Q1T1 stand for the different weight ratios of QSM and NFC: 1:0.25, 1:0.5, 1:0.75, 1:1. Adapted with permission from Baniasadi et al. [39]. Copyright 2021, Elsevier.

For instance, Markstedt et al. [25] optimized and evaluated the printability and biocompatibility of a composite bioink based on NFC and ALG. In this study, a human ear and a sheep meniscus, as models of anatomically-shaped cartilage structures, were successfully 3D bioprinted. The structures showed high shape fidelity and stability after bioprinting, over time. The authors found that NFC/ALG with a concentration of 70/30 (*w*/*w*) showed the highest compressive stiffness (~250 kPa). By further increasing the ALG concentration, the mechanical properties were compromised. Moreover, human nasoseptal chondrocytes (hNSCs) were embedded into the NFC/ALG composite bioink. However, the results demonstrated that the shear forces as well as the crosslinking process negatively affected cell viability. Indeed, cells gradually lost viability, due to the poor diffusion of O_2_/gas, nutrients and growth factors (GFs). To overcome this issue, Ávila et al. [31] printed an hNSC-laden custom-made auricular construct with an open porosity. Importantly, the authors found that NFC/ALG bioink induced the redifferentiation of human chondrocytes. NFC/ALG/HA and human-derived induced pluripotent stem cells (iPSCs) or irradiated human chondrocytes were also studied by Nguyen et al. [32], who used the cell-laden composite bioink for CTE. In particular, NFC mimicked the bulk collagen matrix while ALG stimulated proteoglycans and HA served as a substitute of the one naturally present in the native cartilage. The bioink maintained the pluripotency of the stem cells and induced the development of new cartilage with ECM deposition and high cell density maintenance. ALG sulfate, with the potential to support the chondrocyte phenotype, has also been used in combination with NFC, as a suitable bioink for cartilage bioprinting [33]. The composite bioink promoted bovine chondrocytes (BCs) functions, by inducing collagen II deposition. However, even in this case, cell proliferation was negatively influenced by the bioprinting process. Indeed, small diameter nozzles combined with high extrusion pressures significantly compromised the cells. Currently, the NFC/ALG composite ink formulation has been commercialized with the brand named CELLINK^®^ and has been tested in vitro and in vivo with human chondrocytes and iPSC cells derived from chondrocytes [41,42]. Henriksson et al. [41] successfully formulated a bioink composed of NC and HA (CELLINK-H) for 3D bioprinting in adipose tissue engineering, demonstrating its advantage above the conventional 2D culture system in terms of adipogenic differentiation potential and phenotype maintenance. Moreover, recently, a clinical study on CELLINK^®^ bioink has also been performed in mice [42]. Results have shown that the 3D-bioprinted scaffolds have excellent structural integrity, shape fidelity, and suitable mechanical behavior after 60 days of implantation (with an increase in the compressive stress from 14.9 to 46 kPa). Furthermore, the scaffolds promoted cartilage synthesis. Accordingly, Apelgren et al. [35] described a method for creating human cartilage in vivo by combining human chondrocytes and human mesenchymal stem cells (hMSCs) with a 3D-bioprinted hydrogel scaffold produced using NFC and ALG as the bioink. Immediately following bioprinting, the scaffolds were subcutaneously implanted in mice. The explanted constructs showed a good proliferation ability and ECM deposition after 60 days. The bioprinted cell-laden NFC/ALG scaffold was also studied for its in vivo chondrogenesis potential in a co-culture (hNCs/human bone marrow-derived MSCs (hBMSCs)) system, reporting satisfactory results [42]. An MSC-containing NC/ALG/HA formulation was demonstrated to be an excellent bioink for the development of 3D-bioprinted scaffolds for CTE by Lafuente-Merchan et al. [38]. The authors also evaluated the degradation behavior of the 3D printed scaffolds by measuring the area before and after the immersion in culture medium at selected time points. The NC/ALG and NC/ALG/HA scaffolds decreased the area during the first day. The presence of HA induced a lower area reduction. After 16 days, NC/ALG scaffolds showed a higher total area reduction (22.94%) compared to the NC/ALG/HA ones (20.10%). This effect can be ascribed to the differences in the water swelling ability, which is responsible for higher degradation rates [38]. An optimized NC/ALG bioink was also bioprinted with hNSCs by Jessop et al. [36]. NC particles were produced in the form of a water-based slurry obtained from raw wood-chip biomass. The bioink demonstrated a shear thinning profile with reversible stress softening behavior, which ensured the shape fidelity after the post-printing phase. Human chondrocytes showed a rounded morphology at a longer culture time with high metabolic activity. However, the 3D-bioprinted constructs themselves did not show appropriate mechanical properties with the Young’s modulus of 52.6 kPa. To improve the mechanical properties of the scaffolds, a hybrid bioink was synthesized by Fan et al. [37] through mixing CNC and a Gelatin methacryloyl/methacrylated HA (GELMA/HAMA) solution. CNC was used as the structural support and GELMA/HAMA as the hydrogel carrier for mouse chondrogenic cells (ATDC5). Due to the intrinsic presence of Arginyl-Glycyl-Aspartic acid (RGD sequence) tripeptide in GELMA, the bioink promoted cellular attachment, spreading and differentiation. In conclusion, the hardest component of the bioink (CNC) contributed to conferring good printability. At the same time, the softest component of the bioink (GELMA/HAMA) provided a cell-friendly environment for cell proliferation. From a biological point of view, ATDC5 vitality was not affected during the bioprinting process. Cells remained viable, reaching 99.1% viability after seven days of culture. Recently, Baniasadi et al. [39] designed a viscoelastic bioink based on quince seed mucilage and NFC (QSM/NFC) for direct ink writing. NFC was introduced as a supporting agent to improve the mechanical and rheological behavior of QSM. Indeed, the QSM/NFC inks enabled a good printing fidelity with optimized shape stability and appropriate porosity after freeze-drying. To this aim, the printability of the composite hydrogel was suitably optimized (Figure 2). The apparent viscosity, as a function of the shear rate, showed a shear thinning behavior, with a decrease of three orders of magnitude by increasing the shear rate from 0.01 to 100 s. However, it ranged between the values of neat QSM (Q1T0) and neat NFC (Q0T1).

The blend allowed improving the water swelling and mechanical properties. Indeed, the compressive and elastic moduli of the samples, with the highest NFC content, were both increased by ~100% (from 5.1 kPa and 32 kPa to 10.7 and 64 kPa, respectively). These values ideally match those reported for soft tissues [43]. The degradation rate was assessed by measuring the mass loss during the first 7 days. All the hydrogels highlighted the highest degradation after the first day, which gradually decreased up to 7 days. In particular, the scaffolds did not change the shape over time, with a maximum degradation rate of about 11%. This loss can be mainly attributed to the degradation of QSM as NFC cannot be degraded in the body. By increasing the NFC amount, the degradation rate decreased from 10.6% (Q1T0.25) to 6.4% (Q1T1) [39]. The cell compatibility was evaluated by employing human liver cancer cells (HepG2). The results confirmed no cytotoxicity, good cell adhesion, vitality and proliferation [39].

Even though NC has been widely employed, further studies need to be carried out in terms of functionalization to synthesize innovative and more reliable bioinks for CTE. For example, surface-modified CNC, employed as a green and biocompatible reinforcing agent of a COL hydrogel, could be a promising bioink in this field. The nanocomposite aldehyde-functionalized CNC (a-CNC)/COL (Type I) hydrogel, crosslinked by dynamic Schiff base bonds, showed shear thinning, self-healing, as well as improved mechanical behavior. Moreover, MSCs, encapsulated in the a-CNC/COL hydrogel, highlighted good cell viability after extrusion in vitro. In vivo experiments proved that a-CNCs/COL hydrogel was able to protect MSCs during the injection fitting into the cartilage defect [44].

Another biocompatible polymer, commonly employed in regenerative medicine, is methylcellulose (MC), thanks to its high hydrophilicity, which has a key importance for delivering nutrients to cells [45]. Differently from cellulose, MC is soluble in water solutions, due to the presence of methoxy groups within MC [46]. For this reason, MC can act as a “sacrificial material” for printing. Indeed, recent studies reported the use of MC as a supportive biomaterial to enable biofabrication [47,48]. In CTE, MC was successfully combined with ALG and primary BCs to produce a bioink, which showed good rheological and mechanical as well as interesting biological properties [40].

#### 2.1.2. Marine Algae: Alginate, Agarose, Carrageenan

Algae-based materials, which can be extracted from brown and red *algae*, represent another important group of naturally-derived biopolymers from marine biomass for the design of 3D printed bioink formulations.

##### Alginate

ALG is mainly obtained from *Laminaria hyperborean*, *Laminaria digitate*, *Laminaria japonica*, *Ascophyllum nodosum*, and *Macrocystis pyrifera*. Structurally, it consists of β-D-mannonic acid and α-L-guluronic acids, which are linked through the 1,4-glycosidic bond [49]. Extensive studies using ALG-based hydrogels have described these biomaterials as valuable therapeutic platforms for applications in CTE [49]. ALG is biodegradable since it slowly dissolves in the body due to the release of the crosslinking agents as a consequence of the exchange reactions with the monovalent cations present in the body fluids. ALG is a highly biocompatible biomaterial with tunable viscosity and high hydrophilicity. For this reason, it has been widely employed as a scaffold to load cells and drugs [50]. From a biological point of view, several papers demonstrated that chondrocytes from different sources could maintain a proper differentiated morphology in ALG in vitro [49]. Moreover, they can deposit ECM, which can be found in the ALG itself and in the culture medium [51]. Surprisingly, ALG is useful not only to maintain a differentiated chondrogenic phenotype, but also to restore it in dedifferentiated cells [52] and even in diseased chondrocytes [53]. In vivo, embedded chondrocyte–ALG systems showed a cartilage structure formation [54]. Table 2 summarizes the most relevant works in the 3D printing of cartilage using ALG.

**Figure 3:**
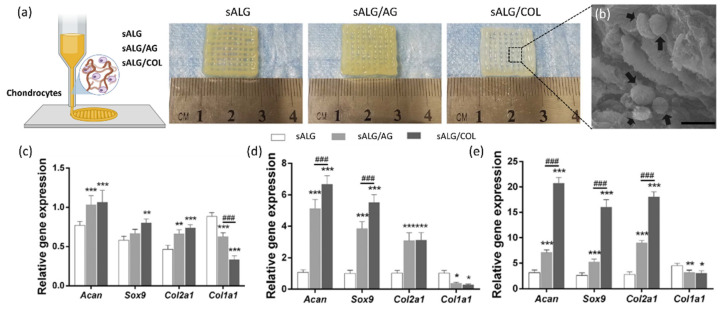
(**a**) Schematic representation of 3D printing (**left**), images of 3D printed sodium alginate (sALG), sALG/Agarose (sALG/AG) and sALG/Collagen (sALG/COL) scaffolds seeded with chondrocytes (**right**). (**b**) Representative scanning electron microscopy image of sALG/COL scaffolds with chondrocytes after 3 days of in vitro culture (Scale bar: 20 μm, the arrows indicated adhered cells). qRT-PCR quantitative analysis of Acan, Sox9, Col2a1 and Col1a1 at (**c**) 3, (**d**) 7 and (**e**) 14 days of cell culture. Dates were reported as the mean ± standard deviation (*n* = 3). (* *p* < 0.05, ** *p* < 0.01 and *** *p* < 0.001 vs. control values, ### *p* < 0.001 indicates the significant difference). Adapted with permission from Yang et al. [58]. Copyright 2018, Elsevier.

For example, Daly et al. [55] successfully printed 3D neat ALG scaffolds. The authors showed that the ALG bioink was able to induce the development of hyaline-like cartilage tissues by using MSCs. However, in 3D bioprinting, by using ALG, structurally simple objects, with limited height, have been produced. For this reason, Armstrong et al. [56] used a synthetic polymer, Pluronic F127, as a sacrificial template, in combination with sodium ALG (sALG), to print 3D structures embedding hMSCs. After printing, the F127 was dissolved and it diffused out of the printed scaffold. The 3D printing compatibility of the bioink was assessed by comparing different F127 concentrations. The best printing behavior was observed using 13% (*w*) F127 with 6% (*w*) sALG, which led to smooth printed struts and reproducible geometries. Lower F127 content bioinks (11% (*w*)) did not successfully gel, leading to a collapsed structure, while higher F127 concentrations (15% (*w*)) produced thicker fibers. Schutz et al. [57] described an ALG/MC blend suitable for the bioprinting of MSCs-laden 3D structures. The printability of the material was ensured by the temporary addition of MC to a 3% (*w*) ALG solution, resulting in improved viscosity, which enabled a precise and easy deposition and high elasticity and stability. The embedded cells showed high viability after 3 weeks in vitro. In 2018, COL type I and agarose (AG) were, respectively, mixed with sALG to produce a bioink, which embedded chondrocytes to obtain in vitro 3D printed cartilage tissue [58]. The bioink showed enhanced mechanical strength. The influence of COL or AG on gelling behavior was negligible, demonstrating the advantage of bioinks for 3D printing. However, the inclusion of COL improved cell adhesion/proliferation and increased the expression of cartilage-specific genes such as Acan, Col2al and Sox9 (Figure 3). A lower expression of Col1a1, the fibrocartilage marker, was also detected.

Schwarz et al. [59] developed a chondro-instructive biomaterial for CTE. In this study, an oxidized ALG hydrogel system, alginate-di-aldehyde (ADA), and gelatin (GEL) were investigated. Ionically crosslinked ADA/GEL showed degradation already on the first day in a standard chondrocyte medium. However, the samples continued degrading, highlighting a total weight loss of about 80% after 14 days. The high degradation of ionically crosslinked ADA/GEL can be ascribed to the melting of the GEL component, which is not influenced by the presence of Ca^2+^. The obtained ADA-GEL was demonstrated to promote chondrocyte viability and the formation of the fundamental cartilage-specific markers, suggesting its potential in CTE. Hierarchically organized scaffolds were also produced by using ADA/GEL hydrogel [26]. Compared to previously reported ADA/GEL compositions, the authors introduced a modified formulation characterized by increased amounts of thermally modified GEL (80 °C for 3 h). Scaffolds were printed in hierarchical complex geometries, with a height of 1 cm. The study demonstrated the possibility to improve the printability of ADA/GEL hydrogels, in terms of shape stability and fidelity, without changing the hydrogel chemistry with the use of additives/crosslinkers. In the literature, there is evidence that ionically crosslinked ALG features low mechanical properties and long-term stability due to ion exchange. For these reasons, Chu et al. [60] developed a double crosslinked ALG (DC-ALG) bioink for 3D bioprinting. The study demonstrated that human umbilical cord MSCs could differentiate into chondrocytes after 4 weeks of culture. In particular, the authors sequentially modified ALG with L-cysteine and 5-norbornene-2-methylamine and crosslinked scaffolds were obtained using CaCl_2_ and ultraviolet (UV) light. The structures showed stronger mechanical properties, similar to native cartilage, good cell viability and chondrogenic potential, indicating cartilage tissue development [60]. DC-ALG showed a prolonged stability (4 weeks), preserving 60% of its original weight after 30 days. Thanks to the double crosslinking, the authors extended the hydrogel degradation time, which is important for CTE. Indeed, numerous studies have highlighted that MSCs employ 4 weeks to differentiate into chondrocytes [60]. Recently, a composite tripolymeric hydrogel bioink for CTE was optimized by Sathish et al. [61]. In particular, the bioink made of GEL, carboxymethylcellulose and ALG, with and without MG63-osteosarcoma cells, was employed to produce a negative meniscus mold. The scaffold soaked in simulated body fluid gradually degraded compared to the phosphate-buffered saline solution. However, the authors observed a complete geometry change after 22 days [61]. From a biological point of view, an increase in terms of cell proliferation and collagen deposition, which affected the biomechanical behavior, was observed, suggesting the obtained 3D-bioprinted scaffold’s suitability for CTE. By coupling 3D printing and impregnating techniques, Sadeghianmaryan et al. [62] developed a hybrid CS/ALG scaffold with nano hydroxyapatite (nHAp). The inclusion of nHAp increased the mechanical properties of scaffolds. The authors also analyzed the degradation behavior (28 days) of different scaffolds with distinct pore sizes (2 and 3 mm). CS scaffolds with the smaller pore size degraded faster. Due to the resistance of nHAp to degradation, nanocomposite scaffolds degraded slower than neat ones. The presence of ALG increased the degradation rate. CS/nHAp impregnated with ALG showed a lower degradation rate than CS scaffolds infiltrated with ALG/nHAp [62]. Furthermore, the live/dead assay highlighted that nHAp had a positive effect on ATDC5 cell adhesion and viability. Finally, CS inclusion provided antibacterial properties, further increased thanks to the presence of nHAp.

##### Agarose

AG is extracted from red seaweed (sea kelp) and has attracated interest in marine plant-derived biomaterials owing to its ability to be produced in the form of a thermal-reversible gel [64]. It has been widely employed in biomedical applications due to its tailored mechanical and self-gelling properties and non-immunogenic features. Furthermore, owing to its stiffness and functional groups, it can improve cellular behavior. Indeed, its water adsorption ability provides the cells with an appropriate microenvironment for their activity [65]. Structurally, AG consists of alternating β-D-galactose and 3,6-anhydro-L-galactose units of agarobiose. It has been applied in the design of drug delivery platforms [66,67], but also for tissue engineering and regenerative medicine [68,69,70,71] in particular, in CTE strategies. In this field of interest, AG-based hydrogels have been employed to assess the mechanical load reaction of chondrocytes and MSCs [65], to maintain the chondrocyte phenotype and to increase cartilage ECM deposition [72,73]. However, a few studies have been focused on the bioprinting of AG blends or neat AG (as indicated in Table 2, previously reported) [73]. In their comparative study, as stated above, Daly et al. [55] found that, overall, AG hydrogels supported the development of hyaline-like cartilage. However, to enhance cellular activity, AG has been combined with other natural polymers, even though these materials have not been 3D printed; however, they could be considered as interesting bioinks. Among them, fibroin/AG blends were shown as a promising biomaterial for CTE. In this context, human elastic cartilage-derived chondrocyte (HECDC) embedded fibroin/AG hydrogels were used to develop a bioactive scaffold [74]. Furthermore, Singh et al. [74] blended AG with silk for CTE. Silk is a natural protein polymer found in the glands of arthropods. It presents considerable mechanical properties and it is produced in fibers by spiders and silkworms. Its amino acid side chains can be chemically modified to improve surface properties or to immobilize GFs. In order to modify silks with specific features, such as the cell/material interaction, different molecular engineering strategies have been followed [75]. However, the use of this material has been limited by the difficulty of developing suitable bioinks because most of them require toxic chemical crosslinkers. In the study by Singh et al. [74], the authors demonstrated that the silk/AG scaffolds preserved the chondrogenic phenotype by increasing sulfated glycosaminoglycans (sGAG) and collagen deposition. Bonhome-Espinosa et al. [76] synthesized a fibrin/AG hydrogel with magnetic features for CTE. The authors noticed that the inclusion of magnetic particles increased the mechanical properties. Moreover, chondrocytes deposited type II collagen, demonstrating a high aptitude of magnetic hydrogels to regenerate hyaline cartilage-like tissue. AG has also been combined with HA, benefiting from its intrinsic biocompatibility and its presence in the native ECM. Indeed, in the study by Choi et al. [77], the HA/AG hydrogel was proposed to overcome the drawbacks of each hydrogel component. HA provided an enhanced matrix microenvironment, while AG contributed in improving the mechanical properties. The composite biomaterial exhibited applicability for CTE. Recently, Gu et al. [63] used carboxylated AG-based bioink for the 3D printing of scaffolds with high stiffness at a physiological temperature. By blending modified AG with small amounts of unmodified AG, they developed a bioink formulation suitable for printing with process parameters. The ink showed a reproducible sol-gel transition at 37 °C. The authors printed anatomically-relevant architectures with high print resolution, size fidelity and high stability over a wide temperature range of 4 °C to 37 °C. Studies with hNCs showed that the bioink could embed a high cell density without altering the printability. Furthermore, printed cells were highly viable and underwent mitosis, an important step of the remodeling process.

##### Carrageenan

Carrageenan (CAR) is a linear sulfated polysaccharide derived from the *Rhodophyceae*, members of red *algae* seaweeds. Three major types of CAR can be cited: iota-CAR (i-CAR), kappa-CAR (k-CAR) and lambda-CAR (λ-CAR). CAR is made by a disaccharide repeating unit which consists of alternating 3-linked β-d-galactopyranose or 4-linked α-d-galactopyranose or 4-linked 3,6-anhydro-α-D-galactopyranose with ester sulfates which emulate the structure of mammalian GAGs [78]. It can be both thermally and ionically crosslinked. It can be combined with other materials such as poly(oxyalkylene amine) [79], methacrylic anhydride [80] and nanosilicates to produce a printable bioink [81] and enable the development of crosslinkable scaffolds. The Young’s modulus of CAR, similarly to AG, depends on the concentration (from 0.10 MPa, 1% (*w*)) to 0.66 MPa, 3% (*w*)), similar to that of native cartilage. Furthermore, the mechanical behavior becomes unpredictable as the water content increases [82]. CAR-based bioinks have demonstrated non-toxicity and their support of the cell/material interaction. The presence of the sulfated backbone mimics naturally occurring sGAGs in cartilage ECM and has demonstrated chondrogenic potency [83,84]. Furthermore, CAR has demonstrated several potential bioactive activities, including antioxidant, antiviral, antibacterial, anticancer, anticoagulant and immunomodulatory ones [85,86,87]. Its chemical structure justifies its employment in drug delivery systems mainly for three reasons [88,89,90]: (1) the presence of glycosidic bonds, which allow it to be broken down by hydrolase enzymes, resulting in high biodegradability, (2) the anionic sulfate groups that confer the polyelectrolyte nature and (3) the presence of hydroxyl groups enabling chemical modifications. Several studies focused on the development of CAR-based formulations to fabricate scaffolds for soft tissue regeneration, even though their use in 3D printing applications has not been fully exploited. For example, Popa et al. [83] designed an injectable k-CAR hydrogel. The material was not 3D printed. However, it was employed to supply adipose tissue-derived MSCs (AMSCs) for CTE. The results highlighted that hydrogels could act as an alternative cell delivery system. Embedded adipose stem cells were viable, proliferated and differentiated in chondrocytes. Similarly, Tytgat et al. [91] functionalized k-CAR and GEL with methacrylate and methacrylamide moieties. This process enabled UV crosslinking by employing a photoinitiator. The results highlighted that the mechanical and swelling properties could be adjusted by varying the hydrogel composition as well as the crosslinking method. The in vitro biocompatibility assays indicated a significantly higher cell viability of AMSCs seeded onto the blends when compared to the single component hydrogel. Regarding the 3D printing application for CTE, Table 3 summarizes the main works carried out using CAR in this field.

**Figure 4:**
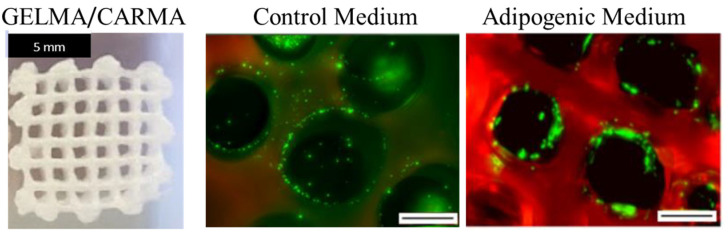
Representative image of 3D printed methacrylamide-modified gelatin (GELMA) and methacrylated k-Carrageenan (GELMA/CARMA) scaffold (**left**); Calcein-acetoxymethyl/propidium iodide staining (**right**) to assess the presence of viable (green fluorescent) and non-viable (red fluorescent) cells at 14 days in control (**middle**) and adipogenic (**right**) medium. Scale bars: 500 μm. Adapted with permission from Tytgat et al. [93]. Copyright 2019, Elsevier.

Wilson et al. [81] introduced an innovative nanoengineered bioink consisting of k-CAR and two-dimensional (2D) nanosilicates (nSi). In particular, by adding nSi, the shear thinning behavior of k-CAR was fine tailored. Furthermore, by tuning k-CAR/nSi ratios, the thermo-reversible gelation of the bioink was modulated to obtain high shape retention properties. Indeed, k-CAR/nSi bioink allowed the obtaining of physiologically relevant scale scaffolds without the use of a secondary support. Li et al. [92] bioprinted multilayered k-CAR/GEL hydrogel scaffolds. The study demonstrated excellent biocompatibility and structural integrity at 37 °C with a compressive modulus of 17.97 kPa. The following strategy allowed the overcoming of the issues related to the use of GEL for biofabrication at 37 °C, without the help of post-crosslinking. In particular, owing to the electrostatic interactions between two opposite charged hydrogels, the mechanical properties at the interface of the 3D printed multilayered scaffold were improved. Successively, Tytgat et al. [93] developed custom-made scaffolds able to support adipose tissue engineering. The scaffolds, made of both GELMA and methacrylated k-CAR (CARMA), were developed using extrusion-based 3D printing. Both types of scaffolds resulted in being stable over time (21 days). Furthermore, they were characterized by high swelling ratios and mechanical properties comparable to those of native tissue (2 kPa) [93]. Concerning the biological behavior, human adipose tissue-derived stem cells (ASCs), seeded onto scaffolds, showed a similar cell viability (>90%) and proliferation rate after 14 days. ASCs differentiated into the adipogenic lineage, although their differentiation potential was lower compared to that of ASCs seeded onto GELMA scaffolds alone (Figure 4) [93].

Kim et al. [94] fabricated ALG/CAR composite scaffolds using extrusion-based 3D bioprinting. Firsty, the authors defined the exact concentration of crosslinking agents by the assessment of the shear modulus of the hydrogels. Indeed, ALG/CAR composite hydrogels were prepared by varying CAR concentrations. Based on the rheological tests, printing resolutions were suitably optimized also by using simulation procedures. The 3D deposition of both hydrogels was assessed and compared with each other by analyzing the shape fidelity. Finally, the cell viability of the 3D printed composite scaffolds was evaluated using live/dead staining and confocal fluorescence imaging. Ilhan et al. [95] aimed at obtaining a stable k-CAR hydrogel bioink for CTE. To this aim, the authors reported a methacrylation process using microwave energy. In particular, the authors synthetized microwave-methacrylated k-CAR (M_w_-CARMA) with a ≥85% degree of methacrylation. Then, M_w_-CARMA was photocrosslinked by UV irradiation for 40 s. Results proved that the hydrogels were characterized by improved mechanical behavior with a lower degradation (~20% at 30 days) than conventional CARMA hydrogels. Viscosities of hydrogels were found to be suitable for 3D bioprinting. Furthermore, they showed enhanced ATDC5 viability, proliferation and GAG deposition. Over recent years, k-CAR has been employed in combination with other synthetic polymers (i.e., polyacrylamide) for CTE [96,97]. However, more extensive studies should be carried out on fully natural k-CAR polymer blends as bioinks for CTE.

### 2.2. Three-Dimensional Printing of Principal ANIMAL-Derived Hydrogels for CTE

#### 2.2.1. Land and Marine-Source Hyaluronic Acid, Collagen, Gelatin

##### Hyaluronic Acid

HA is a polysaccharide which consists of alternating disaccharide units of N-acetylglucosamine and glucuronic acid, linked by β-1,3 and β-1,4 glycosidic bonds [98]. It is naturally present in the mammalian tissue ECM. It is biocompatible and biodegradable, resulting in being totally safe for the human body. Indeed, it can be degraded by a large family of enzymes named hyaluronidases. The easy diffusion of nutrients and wastes and the ability to maintain a hydrated environment makes HA an ideal way for stimulating wound healing [99]. However, HA cannot work as an independent bioink. Indeed, it lacks the mechanical integrity and it has low stability caused by its high-water absorption. Consequently, cells do not adhere to its surface. These disadvantages are often overcome by using other components, in combination, to form a hydrogel suitable for bioprinting [100]. Indeed, in 3D bioprinting, HA has been mainly used as an additive component to improve cell physiological functions through its interaction with binding proteins and cell surface receptors [6]. The HA-based bioinks have been indicated in Table 4.

Park et al. [101] systematically examined the behavior of chondrocytes and osteoblasts cultured within HA and COL type I hydrogels. This study represented a first step toward a bioprinting-based osteochondral tissue regeneration approach. Indeed, the authors demonstrated that hydrogels composed by HA, as the main component, induced a better proliferation of chondroytes. Similarly, hydrogels mainly composed of COL stimulated osteoblasts. This can be scribed to the fact that cells can recognize their native ECM component. Moreover, in the blend, cells located near their native ECM hydrogel migrated toward them. Finally, the authors bioprinted a 3D osteochondral scaffold with two zones, osteoblast-COL hydrogel and chondrocyte-HA hydrogel, and found that viability and functions of respective cell types were ensured up to 14 days. These results suggested that bioprinting can be successfully applied for osteochondral tissue regeneration with the proper choice of hydrogel materials. Subsequently, Costantini et al. [102] demonstrated the possibility of 3D mimetic scaffolds for CTE with a high cell density and printing resolution (≈100 μm) by employing a two coaxial-needles system. In particular, bioinks were developed using cartilage ECM mimetic biopolymers which were suitably modified, namely, GELMA, chondroitin sulfate amino ethyl methacrylate (CS-AEMA) and HAMA. Three photo-crosslinkable bioinks with an increasing level of biomimicry were prepared: GELMA, GELMA + CS-AEMA and GELMA + CS-AEMA + HAMA. Alginate was added to the bioinks as a templating agent to form stable fibers during 3D bioprinting. The bioinks were loaded then with BM SCs. The results demonstrated increased viability and chondrogenic potential, as well as a robust and accurate deposition method, suggesting that this strategy is an interesting candidate for advanced CTE. However, frequently, HA hydrogels have been functionalized to endow specific behavior (i.e., shear thinning) or to improve the mechanical properties and the residence time. The most common functionalization strategies are represented by thiol [105], methacrylate [27], glycidyl methacrylate [106], tyramine [107], or norbornene [108], among several others. Nevertheless, research on HA bioinks is still ongoing as examples of HA bioinks with high shape stability after printing, as well as the optimal long-term formation of cartilaginous tissues, are still rare [109]. For example, when compared to neat HA hydrogels, HAMA is characterized by a longer residence time, maintaining good biocompatibility [110,111,112,113,114]. For this reason, photocrosslinkable HAMA hydrogels have been employed as bioinks to print scaffolds with an increased mechanical stiffness and long-term stability [27]. The authors studied the degradation behavior in phosphate-buffered saline solution with and without hyaluronidase. In general, they found that scaffolds with a lower HA amount degraded faster, due to the lower water uptake. Without enzymatic supplementation, the gel degraded in 14 days. When hyaluronidase was added, the scaffolds lost their integrity within 24 h. Of course, by increasing the concentration, the degradation time increased [27]. When encapsulated in HAMA hydrogels, hMSCs were viable (64.4%) after 21 days in vitro, and the osteogenic differentiation of hMSCs spontaneously occurred at higher HAMA concentrations, without adding osteogenic stimuli [27]. Antich et al. [103] developed a bioink formulation based on HA capable of forming physically crosslinked gels in the presence of calcium. For this reason, ALG was used and it was dissolved in deionized water with HA at concentrations of 2% (*w*/*v*) and 1% (*w*/*v*), respectively. The degradation rate of the bioinks was assessed by evaluating their rheological behavior within 1 month. The compressive and viscoelastic moduli decreased after 1 week, due to the ionic exchange of the Ca^2+^ with Na^+^ in the culture medium and the degradation of HA by hyaluronidases secreted by cells [103]. HA/ALG (1% (*w*/*v*)/2% (*w*/*v*) was found to be the most chondro-permissive formulation. In a recent study, Hauptstein et al [104] designed a novel HA-based bioink for cartilage 3D biofabrication. In particular, a dual-stage crosslinked HA-based bioink enabled the covalent tethering of transforming growth factor-beta 1 (TGF-β1). The bioink showed a high shape fidelity during and after the process. However, the authors employed a low polymer content (2% (*w*)). In vitro, hBMSCs were differentiated in chondrocytes, allowing the deposition of a homogeneous newly-produced ECM and cartilaginous tissue. Furthermore, by analyzed cast and printed bioinks, the authors successfully demonstrated that TGF-β1 was not negatively affected by 3D printing. Altogether, the presented bioink composition bears great potential for future investigation towards CTE. It is necessary to underline that a large amount of HA has been obtained from the marine environment also, mainly from cartilage and from the vitreous humor of different fishes [115]. However, optimized processes for its recovery and production must be developed, and further studies should be carried out to optimize its printability as a bioink for CTE.

##### Collagen and Gelatin

COL is the most hydrophilic prevalent protein in mammals. It includes about 30% of the total mammalian protein mass. These proteins are essentially structural ECM components. They consist of three polypeptide chains, known as α chains, and contain triple helical domains [116]. COL is biodegradabe as it can be broken down by different catabolic processes in the body, involving the enzymolysis of collagenase. After the triple helices are cleaved, non-specific proteinases and gelatinases can continue to degrade the COL molecules. There are 28 different types of COLs characterized by different amounts of triple helices and alternative combinations of α-chains [117]. COL has integrin-binding domains, which promote cell adhesion and proliferation [118]. It does not cause serious immunological responses, even though the immunogenicity of COL is influenced by the presence of other proteins and crosslinking reagents. Animal-derived COL may lead to inflammation and disease transmission [119,120]. COL type I is a member of the fibril-forming subfamily of COLs and is commonly used in bioprinting. However, it is not often used as a neat bioink due to its mechanical instability and slow gelation rate, which limit its ability for self-staining. COL maintains a liquid state below 37 °C [118]. Studies using neat COL as a bioink often aim at improving its mechanical properties by using sacrificial supports which are removed in a post-processing phase [121] or by directly modifying COL bioink properties (i.e., the concentration or crosslinking method). Indeed, different strategies have been investigated to improve the printability of COL bioinks by controlling the gelation kinetics and their storage modulus [122,123]. In particular, an increased storage modulus, exceeding the loss modulus, has been found to correspond to the improved printability of COL bioink. The principal COL-based bioinks used in CTE have been reported in Table 5.

In particular, Osidak et al. [124] formulated a COL solution, branded Viscoll, as a bioink with a high fidelity performance and good printability. Additionally, Diamantides et al. [122] demonstrated that both gelation kinetics and the viscoelastic properties of COL bioinks depended on pH. Indeed, the highest storage modulus was found at pH values of 7.5–8.0 and 8.0–8.5. Outside these ranges it decreased. The study also demonstrated that, by using a blue-light-activated riboflavin crosslinker, it was possible to increase it, negatively affecting the chondrocyte viability (approximately 20%). The same authors found, in a more recent study, that the storage modulus was dependent on the cells density and gelation degree [125]. The storage modulus and viscosity increased with cell density before gelation, but decreased after gelation [125]. COL is often blended with other naturally-derived biomaterials to improve the structural integrity, printability and bioactive properties. As already previously discussed, COL has been combined with NC [44], ALG [58] and HA [134]. A recent study also examined 3D-bioprinted constructs fabricated using ALG/GEL/fibrinogen bioink mixed with hBMSCs. The authors demonstrated the safety of the bio-extrusion and gelation of the bioink at low cell concentrations in terms of MSC metabolism and chondrogenic potential in hypoxic conditions under the effect of TGF-β1 and bone morphogenetic protein 2 (BMP-2) [126].

As with COL, GEL has cell adherence sites (RGD sequences). GEL derives from the COL via hydrolytic degradation. It also shows great biodegradability, low antigenicity and biocompatibility. Indeed, it has been used as a bioink in 3D bioprinting for CTE. However, it has been often combined with CNC [37], ALG [26,59,60,61], k-CAR [92,93], chondroitin sulfate amino ethyl methacrylate and HAMA [101]. Due to its weak mechanical properties and water swelling above 35 °C, GEL can be easily modified to introduce the methacrylate group. In such a way, the photo-crosslinkable GELMA [135,136] has been employed in extrusion printing [137]. In addition, GELMA scaffolds have been designed to have a 100% interconnected pore network in the concentration range of 10–20% (*w*/*v*), mechanical stability and high cell viability (>97%) [137]. Recently, these GELMA hydrogels have been generally combined with other biomaterials to optimize their functions and satisfy tissue engineering requirements. For instance, GELMA supplemented with gellan gum showed to be a promising bioink. In particular, Mouser et al. [127] investigated the suitability of GELMA/gellan gum for bioprinting in CTE. The addition of gellan gum improved the strut deposition, increased the stiffness and supported chondrogenesis. However, high gellan gum concentrations compromised cartilage ECM production and distribution, resulting in very high yield stresses to allow cell embedding. Furthermore, Fan et al. [128] developed a GEL/ALG hydrogel scaffold reinforced with nHAp. The addition of nHAp modified the surface roughness of the scaffolds, which influenced the cell/material interaction and improved their biodegradability. Scaffolds with the highest nHAp concentration (30%) highlighted the lowest degradation within one week. Successively, the weight loss rate increased from the second week [128]. The 3D-bioprinted scaffold showed no cytotoxicity and supported the adhesion and proliferation of mouse chondrocytes. Levato et al. [129], instead, combined multipotent articular cartilage-resident chondroprogenitor cells and MSC-laden GELMA bioink to bioprint a model of articular cartilage. The model was characterized by the presence of defined regions, each with distinct cellular and ECM compositions. These results paved the way to the biofabrication of 3D constructs with multiple cell types for CTE. Another interesting bioink has been developed by Singh et al. [130], who fabricated silk fibroin blends with GEL through physical crosslinking interactions. The authors printed scaffolds with a suitable swelling behavior, optimal rheology and supportive structure. By increasing the content of GAGs and COL, this bioprinted scaffold allowed the chondrocytes growth and proliferation and the upregulation of the chondrogenic genes’ expression. A silk fibroin/GEL-based hydrogel has also been used by Trucco et al. [131]. In the study, the authors encapsulated hMSCs in the tri-composite hydrogel bioink. The results indicated that the cell viability was not affected by the printing process and hMSCs produced cartilaginous ECM. In particular, the cells cultured with TGF-β3 generated a stable chondrogenic phenotype with no evidence of hypertrophy. Indeed, typical anabolic signaling pathways such as Wnt, Notch and HIF-1, which are repsonsible of cartilage repair, were activated. Huang et al. reported the development of GEL/HAp bioink [132]. The study demonstrated that the addition of HAp in the GEL scaffold improved the gelation kinetics, the rheological properties and allowed better control of the 3D printing process. HAp also enhanced the mechanical strength of the scaffold, as shown by the increased compression modulus (from 70.49 ± 0.67 kPa (GEL) to 77.35 ± 0.96 kPa (GEL/HAp)). The scaffold also supported the adhesion, growth and proliferation of human umbilical cord blood-derived MSCs (hUCB-MSCs) and induced their chondrogenic differentiation in vitro. Furthermore, the authors conducted the in vivo studies on a pig model, which resulted in a promotion of the cartilage defect repair.

**Figure 5:**
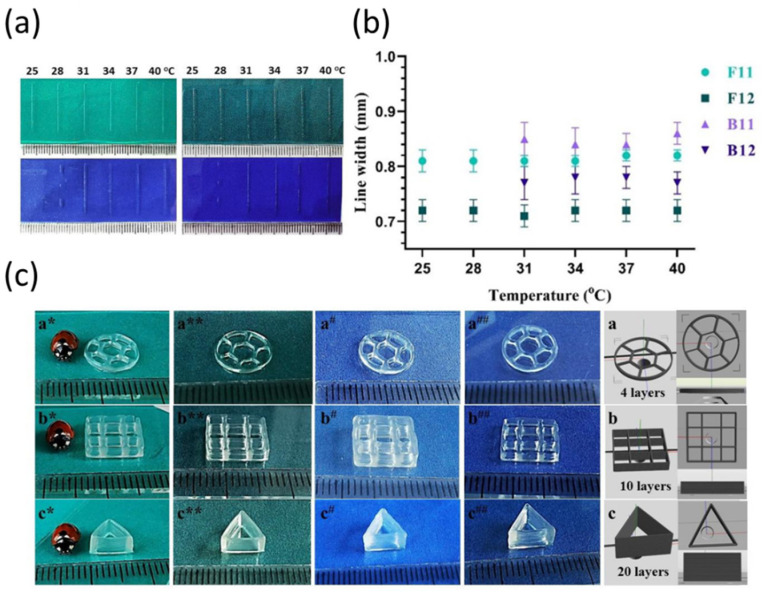
(**a**) Results from of printing tests performed in the temperature range of 25 to 40 °C. (**b**) Measurements of printed line widths vs. temperature. (**c**) Representative 3D printed constructs with F11 (*), F12 (**), B11 (#) and B12 (##) designed as discs with different patterns and geometries. F and B stand for fish and bovine nature, respectively, while 11 and 22 stand for the concentration of methacrylated gelatin (GELMA, % (*w*/*v*)). To prepare all samples, 1.1 NFC% (*w*/*v*) was used. Adapted with permission from Cernencu et al. [133]. Copyright 2021, Multidisciplinary Digital Publishing Institute. * indicates *p* < 0.05.

COL from marine organisms is also an attractive choice, as it is highly abundant in solid marine waste and it has been found in different marine species such as, sponges, corals, salmon, jellyfish, coralline, red *algae*, sea urchins and mollusks [138,139]. It provides fewer risks of disease transmission and religious restrictions than mammalian COL [140]. Indeed, marine COL is characterized by similar a biocompatibility and functionality as the mammalian one, without its limitations. In terms of printability, the mechanical properties limit its applicability. Indeed, concentrations (<5 mg/mL) commonly used for CTE are generally unsuitable for the bioprinting process. Even though increasing the working concentration of COL improves its printability, the scaffold can present high stiffness, which may inhibit cell spreading and proliferation [140]. Another disadvantage of neat COL is the long time needed to allow the gelation that may undermine the smooth layer-by-layer deposition and the structural integrity of the final scaffold. Considering the advantages of marine COL compared to mammalian COL, there have been recent attempts to employ marine COL for bioprinting in CTE. To enhance the printability of these marine COL bioinks, several approaches have been investigated, including the incorporation of an additional supporting polymer and the functionalization of native COL to enable chemical crosslinking [141]. However, a few studies have been focused on the 3D bioprinting of marine COL for CTE. Indeed, different bioinks have been developed by blending blue shark and eel COL, respectively, with ALG, which allowed the bioprinting of constructs with a higher stability and mechanical strength. The scaffolds were further reinforced by the ionic crosslinking of ALG and were mainly used for osteochondral defect repair [142,143]. Similarly, fish skin COL has been introduced by Liu et al. [144] into a bioink formulation containing methacrylated hydroxybutyl CS. The bioink provided a favorable substrate for cell attachment and growth. On the other hand, Sanz et al. [145] synthesized UV crosslinkable red snapper COL via a reaction with methacrylate functional groups. The authors showed that the chemical crosslinking step improved COL’s structural integrity. Recently, the attention on this bioink has grown. Indeed, highly reliable and stable bioinks, based on GELMA derived from fish skin (Fish GEL) and NFC, were designed in 2021 by Cernencu et al. [133]. Fish GEL showed superior thermal stability, further overcoming the issues related to the significant viscosity changes of mammalian GEL upon temperature variation. The authors comparatively studied fish- and bovine-based GELMA formulations by systematically analyzing the printability in physiological conditions (Figure 5).

In particular, acellular hydrogel structures were bioprinted using microvalve-based printing and crosslinked. The structural integrity of printed structures as well as morphological and swelling features were studied. The printability and biocompatibility of the cell-embedded bioink were further assessed in terms of cell viability after printing using hAMSCs, demonstrating high viability and proliferation when Fish GELMA was employed [133]. Similarly, Maher et al. [146] studied the suitability of marine COL for 3D bioprinting and tissue engineering without focusing on CTE. In particular, the authors compared marine COL type I (Macruronus novaezelandiae, Blue Grenadier) with the more established porcine COL type I. Both collagens were methacrylated to allow for UV crosslinking during extrusion 3D bioprinting. The materials were shown to be highly cytocompatible with L929 fibroblasts. The mechanical properties of the marine-derived COL were generally lower than those of the porcine-derived COL; however, the Young’s modulus for both COLs was shown to be tunable over a wide range and was also interesting for CTE. In conclusion, the authors suggested marine-derived COL as a potential candidate in CTE, even though its applicability may be limited due to its lower thermal stability [146].

#### 2.2.2. Chitosan

Among the different marine-derived polysaccharides, CS is also one of the most abundant ones. It is composed of randomly distributed β-(1→4)-linked D-glucosamine (deacetylated unit) and N-acetyl-D-glucosamine (acetylated unit) and it is produced by chitin deacetylation. Typically, it is slightly soluble in water, but at pH < 6.2, it can be dissolved in solutions [147,148]. Furthermore, CS is non-toxic, biodegradable, biocompatible and bio-adhesive. In particular, the degradation by-products are elements involved in the synthesis of cartilage such as chondroitin sulfate, HA, keratin sulfate and glycosylated COL (type II) [149]. However, renewable CS has weak mechanical strength, which limits its use for CTE [147,148,149]. The most important CS-based bioinks for CTE are reported in Table 6.

In a study by Ye et al. [148], the authors showed that human infrapatellar fat pad AMSCs seeded onto 3D printed CS scaffolds were addressed towards chondrogenesis using GFs such as TGF-β3 and BMP-6, with a cartilage-like tissue formation after 4 weeks of culture. He et al. [149] modified CS with ethylenediaminetetraacetic acid (EDTA) before the addition of Ca^2+^ to increase the amount of CS-Ca^2+^ crosslinking. In such a way, the authors obtained good stability and mechanical properties through the adjustment of the two-bioink components, CS and modified CS. Furthermore, the bioink highlighted low cytotoxicity, good cell proliferation, fast gelation and high precision during printing. In another study, a CS bioink was prepared by dissolving CS in an acidic mixture and its properties were analyzed for extrusion printing [149]. Concentrations of CS higher than 11% (*w*) and lower than 4% (*w*) were found to be too viscous and too liquid, respectively. The printed scaffolds, with medium CS concentrations, showed a high resolution, high shape retention and good mechanical properties. Sadeghianmaryan et al. [28] varied the CS concentrations in the range between 8% and 12% (*w*/*v*) and selected 10% for further studies. Results from the mechanical characterization showed the highest elastic modulus for the scaffolds crosslinked with the air-drying technique. However, 10% (*w*/*v*) CS scaffolds showed a degradation of 15% in 7 days. ATDC5 cells cultured on the 3D printed CS scaffolds showed high cell adhesion with a round morphology and high biocompatibility. In conclusion, CS may be a suitable bioink, but often requires additional components to improve its mechanical behavior [28].

## 3. Conclusions and Future Perspectives

Over the past five years, 3D bioprinting has allowed the fabrication of biomimetic scaffolds by the layer-by-layer deposition of a wide range of biomaterials. In this scenario, different bioinks have been developed. In particular, based on the properties of cartilage tissue and healing mode of cartilage defects, different naturally-derived hydrogels have highlighted to be promising due to their interesting features: tailored printability and mechanical strength, intrinsic chondrogenesis induction, biocompatibility and biodegradability. In this context, ideally, scientists would design the scaffolds with the aim to degrade over time concurrently with the formation of new tissue as a consequence of cell proliferation. For this reason, the choice of a suitable bioink, in part, is based on its degradation rate, which, ideally, should mimick the rate of ECM formation. With the advancement of technology, the degradation rate of the scaffolds is adjustable to enable the controlled release of GFs and differentiation factors, which might be included within the desired bioink. Although naturally-derived bioinks should be highly biocompatible, challenges with these bioinks may include host immunological responses in vivo, a low degradation rate, degraded biomaterial toxicity, the interference of cell/cell interactions and low mechanical properties of bioprinted constructs correlated to the degradation, limiting the possibility of such materials in clinical applications. However, research is still ongoing and other innovative bioinks, suitably functionalized by their enrichment with chondrogenic cells and GFs, are still needed to optimize the CTE strategy and promote cartilage repair. Indeed, inspired by nature, bioink properties could be fine-tuned to perfectly recapitulate the 3D microenvironment of the regenerating tissue, in terms of mechanical, morphological and biological features. It is also important to better understand the biodegradability or fate of these 3D printed scaffolds in the body, as this aspect has been barely taken into consideration. Furthermore, even though 3D-bioprinted scaffolds have been highlighted to successfully promote chondrogenesis in vitro, future pre-clinical and clinical studies are still required to demonstrate their ability in vivo. Indeed, to date, 3D-bioprinted CTE scaffolds have yet to be successfully translated into a clinical application. The technique has shown good reproducibility and, also, the possibility to scale-up the process. However, some limitations need to be overcome. Among them, it is important to cite the poor availability of appropriate eco-friendly bioinks, as already previously discussed, but mostly, the high costs and complex regulatory pathways for the approval of scaffolds for CTE (lack of bioprinting specific standards). As widely recognized, the proposed clinical application of bioprinting includes three different phases, strictly coordinated in a reverse engineering approach: (a) medical imaging by computed tomography or magnetic resonance, (b) scaffold design by computer-aided dedicated softwares and (c) scaffold manufacturing by 3D bioprinting. It is worth noting that, before scaffold production, cells would be harvested from the patient and properly embedded into the specific hydrogel ink to obtain the suitable bioink. At this point, the scaffold would be bioprinted and successively crosslinked before implantation into the defect site. The main drawback of this strategy lies in the fact that the scaffold needs to be handled by the surgeon before implantation. An alternative strategy is the use of in situ bioprinting where bioinks can be directly printed into the defect site by the surgeon in a clinical setting. Currently, BioPen, developed by Onofrillo et al., can be considered the only system that has been tested as a surgical procedure in a sheep model using homologous stem cells [150]. In particular, the 3D bioprinting device allowed the in situ 3D bioprinting of cells (hADSCs, harvested from the infrapatellar fat pad of donor patients affected by osteoarthritis) embedded in a hydrogel ink (GELMA and HAMA) in a surgical setting. Given its ability to extrude in a core/shell manner, the Biopen preserved cell viability during the printing process [150].

Recently, 3D bioprinting has evolved towards the next generation of this technology, namely, four-dimensional (4D) bioprinting, even though important limitations of the latter should still be properly addressed. Four-dimensional bioprinted scaffolds are 3D biofabricated structures, in which the shape, properties and functions can change over time when exposed to a determined external stimulus (i.e., temperature, electric/magnetic field, light, pH and ions) [151]. In this context, stimuli-responsive hydrogels are attracting research attention. In CTE, 4D-bioprinted hydrogels could be interesting as they could be able to adapt their shape, structure and function, according to the needs of the cartilage tissue regeneration process. For example, a hydrogel able to change its degradability behavior could be useful to coordinate the tissue ingrowth space. Indeed, a faster degradability could result in insufficient mechanical support, compromising the growth of the neocartilage. However, it is still very difficult to accurately tune the properties of these hydrogels. For instance, Almeida et al. [152] designed a shape-morphing ALG hydrogel to allow the development of a cartilaginous tissue in vitro. To this end, a porous, shape-memory ALG scaffold was produced and decorated with ECM cues to guide MSCs’ differentiation towards chondrocytes. Shape-memory properties were introduced by covalent crosslinking ALG via a carbodiimide reaction. The architecture of the scaffold was modified using a directional freezing technique, which introduced anisotropic-aligned pores. This morphology allowed the improvement of the mechanical properties of the scaffold, to promote higher levels of GAGs and collagen deposition by using hMSCs. The presence of COL increased cell recruitment into the scaffold and allowed cartilage tissue deposition. This hydrogel could be easily exported to 4D bioprinting. However, until now, there have been very few cases of 4D-bioprinted resorbable materials for CTE [153]. Indeed, 4D bioprinting is still in the proof-of-concept phase. The main issues are related to the availability of adequate bioinks. Indeed, in addition to being highly cell-friendly and with optimized shear thinning behavior, the novel hydrogels should have appropriate stiffness and responsiveness to multiple external stimuli, adding, in this way, a higher level of biomaterial complexity. Nevertheless, this technology is opening up a new frontier for the biofabrication community and has shown the potential to fully revolutionize CTE strategies.

## Figures and Tables

**Table 1:** Nanocellulose-based bioinks employed for cartilage tissue engineering.

Bioink	Cell Population	Main Outcomes	Reference
Nanofibrillated Cellulose (NFC)/Alginate (ALG)	Human nasoseptal chondrocytes (hNSCs, 15 × 10^6^ cells/mL)	High shape fidelity; decrease of cell viability due to shear forces during mixing and crosslinking	Markstedt et al. (2015), [25]
NFC/ALG	hNSCs (20 × 10^6^ cells/mL)	Optimized shape and stability at 28 days; neo-synthesis of cartilage-specific extracellular matrix	Ávila et al. (2016), [31]
NFC/ALG/ Hyaluronic acid (HA)	Human-derived induced pluripotent stem cells (iPSCs); Human chondrocytes (20 × 10^6^ cells/mL)	Maintaining of pluripotency of stem cells; cartilage formation; collagen expression	Nguyen et al. (2017), [32]
NFC/ALG sulfate	Chondrocytes from old calves (6 × 10^6^ cells/mL)	High viability of chondrocytes; deposition of collagen II; wide diameter, conical needles preserved cell function	Müller et al. (2016), [33]
NFC/ALG	Human bone marrow–derived mesenchymal stem cells (hBMSCs) and hNSCs (10 × 10^6^ cells/mL)	Good printability and dimensional stability; good mechanical properties; chondro-permissive; glycosaminoglycan (GAG)-positive cell proliferation	Möller et al. (2017), [34]; Apelgren et al. (2017), [35]
NC/ALG	hNSCs (2 × 10^6^ cells/mL)	Shear thinning behavior; favorable swelling features; high metabolic activity of hNSCs; limited mechanical properties	Jessop et al. (2019), [36]
Cellulose nanocrystals/Gelatin methacryloyl/ methacrylated hyaluronic acid (CNC/GELMA/HAMA)	Mouse chondrogenic cell line (ATDC5, 1 × 10^6^ cells/mL)	Good printability; shear thinning behavior; high structural support and integration; good cell viability	Fan et al. (2020), [37]
NC/ALG/HA	Murine D1-MSCs (2.5 × 10^6^ and 5 × 10^6^ cells/mL)	HA induced a more fibrous structure; less rounded morphology; earlier water swelling in 3 to 4 h; slower degradation; better biological behavior	Lafuente-Merchan et al. (2021), [38]
Quince seed mucilage (QSM)/NFC	Human liver cancer cells (HepG2, 5 × 10^6^ cells/mL)	Precise control on printing fidelity; suitable water uptake capacity and mechanical properties; good cell attachment and proliferation	Baniasadi et al. (2021), [39]
Methylcellulose (MC)/ALG	Primary bovine chondrocytes (BCs, 0.5 × 10^6^ cells/mL)	Good viscosity and stability; high cell survival and proteoglycan matrix production	Hodder et al. (2019), [40]

**Table 2:** Alginate and Agarose-based bioinks employed for cartilage tissue engineering.

Bioink	Cell Population	Main Outcomes	Reference
Pure Alginate (ALG) Pure Agarose (AG)	Human bone marrow stromal cells (hBMSCs, 2 × 10^6^ cells/mL)	Development of hyaline-like cartilage tissue	Daly et al. (2016), [55]
Pluronic F127/ALG	hMSCs (3 × 10^6^ cells/mL)	Increased shear thinning; good compressive modulus; good cell viability over 10 days and chondrogenic properties over five weeks	Armstrong et al. (2016), [56]
ALG/Methylcellulose (MC)	hBMSCs (5 × 10^6^ cells/g)	Enhanced viscosity; high elasticity and stability; enhanced microporosity; high viability; maintenance of differentiation potential	Schutz et al. (2017), [57]
Collagen (COL)/ALG, AG/ALG	Chondrocytes (1 × 10^7^ cells/mL)	Improved mechanical strength; better cell adhesion; increased cell proliferation; increased cartilage genes expression; lower expression of Col1a1	Yang et al. (2018), [58]
Oxidized alginate-di-aldehyde (ADA)/ Gelatin (GEL)	Human nasoseptal chondrocytes (hNSCs, 4 × 10^6^ cells/mL)	Open inner structure; high viscosity and shear thinning behavior; promotion of collagen type II and cartilage proteoglycans Enhanced printability; high shape stability and fidelity without use of chemical additives or crosslinkers	Schwarz et al. (2020), [59] Kreller et al. (2021) [26]
Double crosslinked ALG (DC-ALG)	Human umbilical cord MSCs (huMSCs, 1 × 10^5^ cells/mL)	Strong mechanical properties; better stability; good cell viability; high printing accuracy (∼200 µm); expression of chondrogenic genes	Chu et al. (2021), [60]
GEL/Carboxymethyl cellulose/ALG	Osteosarcoma cells, MG63	Increased collagen deposition; improved cell proliferation	Satish et al. (2022), [61]
ALG/CS/ Hydroxyapatite (nHAp)	Chondrocytes (ATDC5, 2 × 10^5^ cells/mL) (top seeding)	Increased elastic modulus; improved cell attachment and viability; antibacterial ability of CS	Sadeghianmaryan et al. (2022), [62]
Carboxylated AG/neat AG	hNCs (3 × 10^7^ cells/mL)	High print reproducibility and size fidelity; high stability over a wide temperature range (4–37 °C); high cell density without impact on printability	Gu et al. (2020), [63]

**Table 3:** Carrageenan-based bioinks employed for cartilage tissue engineering.

Bioink	Cell Population	Main Outcomes	Reference
kappa Carrageenan/Nanosilicates (k-CAR/nSi)	Mouse preosteoblasts (MC3T3-E1)	High shape and structural fidelity; enhanced mechanical properties	Wilson et al. (2017), [81]
k-CAR/Gelatin (GEL)	Mouse myoblasts (C2C12, 2.8 × 10^5^ cells/mL	Good multilayered structural stability at 37 °C and a high cell viability	Li et al. (2019), [92]
Methacrylamide-modified gelatin (GELMA)/methacrylated k-CAR (CARMA)	Human adipose tissue-derived stem cells (hASCs, 1 × 10^5^ cells) (top seeding)	Good stability; high water swelling; mechanical properties comparable to those of native tissue	Tytgat et al. (2019), [93]
k-CAR/Alginate (ALG)	Rabbit adipose mesenchymal stem cells (AMSCs, 5 × 10^5^ cells/mL)	Excellent structural strength and printability without significant negative effects on cell viability	Kim et al. (2019), [94]
CARMA	Embryonal carcinoma-derived chondrogenic cells (ATDC5, 2 × 10^7^ cells/mL)	Improved mechanical behavior and degradation time; improved cell migration, proliferation and differentiation	Ilhan et al. (2020), [95] [95]

**Table 4:** Hyaluronic acid-based bioinks employed for cartilage tissue engineering.

Bioink	Cell Population	Main Outcomes	Reference
Hyaluronic acid (HA)	Chondrocytes (2 × 10^6^ cells/mL)	High viability and function of cells maintained up to 14 days of culture; cell migration	Park et al. (2014), [101]
Methacrylated hyaluronic acid (HAMA)	Bone marrow stromal cells (BMSCs, 1 × 10^7^ cells/mL)	Enhanced viability; cell chondrogenic differentiation potential; high mechanical properties; high resolution of the deposition method; resistant to degradation; good biocompatibility	Costantini et al. (2016), [102]
HAMA	hBMSCs (2 × 10^6^ cells/mL)	Increased mechanical stiffness; long-term stability; high cell viability; spontaneous osteogenic potential	Poldervaart et al. (2017), [27]
HA/ALG	Chondrocytes (1 × 10^7^ cells/mL)	Good printability; gelling abilities; stiffness and good degradability; high cell viability	Antich et al. (2020), [103]
Covalently tethered TGF-β1/HA	hBMSCs (2 × 10^6^ cells/mL)	High shape fidelity; highly porous network with low polymer content (2% (*w*)); high chondrogenisis; homogeneous ECM distribution	Hauptstein et al. (2021), [104]

**Table 5:** Collagen-based bioinks employed for cartilage tissue engineering.

Bioink	Cell Population	Main Outcomes	Reference
COL	Bovine primary articular chondrocytes (bPAC, 10^7^ cells/mL)	Increased storage modulus and improved printability by blue-light-activated riboflavin crosslinker; gelation kinetics and storage moduli pH dependent	Diamantides et al. (2017), [122]
Collagen (COL) branded Viscoll (Imtek Ltd., Russia)	Mouse fibroblasts expressing green fluorescent protein (NIH 3T3-GFP, 0.5 × 10^6^ cells/mL)	Increased storage modulus; improved printability of collagen; appropriate support of spatial distributuin of tissue spheroids into rigid patterns with resolution of 0.5 mm; sufficient cell viability	Osidak et al. (2019), [124]
COL	bPAC, up to 10^8^ cells/mL	Increased storage modulus and viscosity before gelation; storage modulus after gelation and gelation rate decreased along with increasing cell density	Diamantides et al. (2019), [125]
Alginate (ALG)/Gelatin (GEL)/Fibrinogen	Mesenchymal stem cells (MSCs, 1–2 × 10^6^ cells/mL)	Hypoxia prevention of calcifications by hypoxia; enhanced chondrogenesis by TGF-β1/3 combined with BMP-2	Henrionnet et al. (2020), [126]
Gelatin methacryloyl (GELMA)/Gellan gum	Equine primary chondrocytes (1–2 × 10^7^ cells/mL)	Improved filament deposition; increased construct stiffness; chondrogenic potential	Mouser et al. (2016), [127]
GEL/ALG/nano-hydroxyapatite (nHAp)	Mouse chondrocytes (2 × 10^5^ cells/mL)	Improved surface roughness and biodegradability; no cytotoxicity; enhanced cell adhesion and growth; high cell viability	Fan et al. (2019), [128]
GELMA	Multipotent articular cartilage-resident chondroprogenitor cells (ACPCs), MSCs (1.5 × 10^7^ cells/mL)	MSCs-laden GELMA printable in a zonal-like architecture; biomimetic GAG distribution	Levato et al. (2017), [129]
Silk/GEL	Chondrocytes (10^6^ cells/mL)	Suitable swelling behavior; optimal rheology; supportive structure; cartilage ECM formation; chondrogenic phenotype maintenance	Singh et al. (2019), [130]
Silk Fibroin/GEL	hMSCs (0.6 × 10^7^ cells/mL)	Printing parameters optimized by the model; good chondrogenicity	Trucco et al. (2021), [131]
GEL/HAp	Human umbilical cord blood-derived MSCs (hUCB-MSCs, 10^5^ cells) (top seeding)	Cell adhesion and proliferation support; chondrogenic differentiation induction; increased hydrogel fluidity; improved gelation kinetics and rheological properties	Huang et al. (2021), [132]
NFC/Fish GELMA NFC/Bovine GELMA	Human adipose tissue-derived MSCs (hAMSCs, 10^6^ cells/mL)	Good printability; high shape fidelity and well-defined internal structure; Fish GEL exhibited a broader bioprintability window; NFC/GELMA allowed cell growth and proliferation	Cernencu et al. (2021), [133]

**Table 6:** Chitosan-based bioinks employed for cartilage tissue engineering.

Bioink	Cell Population	Main Outcomes	Reference
CS	Infrapatellar fat pad AMSCs (7.5 × 10^5^ cells/mL)	Cartilage-like tissue formation in 4 weeks of culture	Ye et al. (2014), [148]
Carboxymethyl CS	Rabbit chondrocytes (1 × 10^5^ cells/mL)	Higher storage and loss moduli; low cytotoxicity; good cell proliferation rate; fast gelation; high printability	He et al. (2020), [149]
CS	Mouse chondrogenic cell line (ATDC5, 10^6^ cells/mL)	Higher elastic modulus for scaffolds with smaller pore sizes; high cell adhesion	Sadeghianmaryan et al. (2020), [28]

## Data Availability

Not applicable.

## Abbreviations

**GENERAL**	
RGD	Arginyl-Glycyl-Aspartic acid
BMP-2	Bone morphogenetic protein 2
CTE	Cartilage tissue engineering
ECM	Extracellular matrix
4D	Four dimensional
GFs	Growth factors
O_2_	Oxygen
3D	Three dimensional
TGF-β1	Transforming GF beta 1
UV	Ultraviolet
**MATERIALS**	
AG	Agarose
a-CNC	Aldehyde-functionalized cellulose nanocrystals
ALG	Alginate
ADA	Alginate-di-aldehyde
CAR	Carrageenan
CNC	Cellulose nanocrystals
CS	Chitosan
CS-AEMA	Chondroitin sulfate amino ethyl methacrylate
COL	Collagen
DC-ALG	Double crosslinked Alginate
GEL	Gelatin
GELMA	Gelatin methacryloyl or Methacrylamide-modified Gelatin
HA	Hyaluronic acid
i-CAR	Iota-Carrageenan
k-CAR	Kappa-Carrageenan
λ-CAR	Lambda-Carrageenan
HAMA	Methacrylated Hyaluronic acid
CARMA	Methacrylated k-Carrageenan
MC	Methylcellulose
M_w_-CARMA	Microwave-methacrylated k-Carrageenan
NC	Nanocellulose
NFC	Nanofibrillated cellulose
nHAp	Nano-Hydroxyapatite
nSi	Nanosilicates
QSM	Quince seed mucilage
sALG	Sodium Alginate
**CELLS**	
AMSCs	Adipose tissue-derived mesenchymal stem cells
bPAC	Bovine primary articular chondrocytes
hASCs	Human adipose tissue-derived stem cells
hBMSCs	Human bone marrow–derived mesenchymal stem cells
HepG2	Human liver cancer cells
hMSCs	Human mesenchymal stem cells
hNSCs	Human nasoseptal chondrocytes
hUCB-MSCs	Human umbilical cord blood-derived mesenchymal stem cells
huMSCs	Human umbilical cord mesenchymal stem cells
iPSCs	Human-derived induced pluripotent stem cells
ATDC5	Mouse chondrogenic cell line
NIH 3T3-GFP	Mouse fibroblasts expressing green fluorescent protein
C2C12	Mouse myoblasts
MC3T3-E1	Mouse preosteoblasts
ACPCs	Multipotent articular cartilage-resident chondroprogenitor cells
BCs	Primary bovine chondrocytes
